# Supplementary material for: Complex‐centric proteome profiling by SEC‐SWATH‐MS
Source: Mol Syst Biol. 2019 Jan 14;15(1):e8438. doi: 10.15252/msb.20188438 (PMC6346213; doi:10.15252/msb.20188438)
Supplement: Supplementary file 8 — Dataset EV7 [file MSB-15-e8438-s008.zip › feature_plots_string/Q96EH5.pdf]

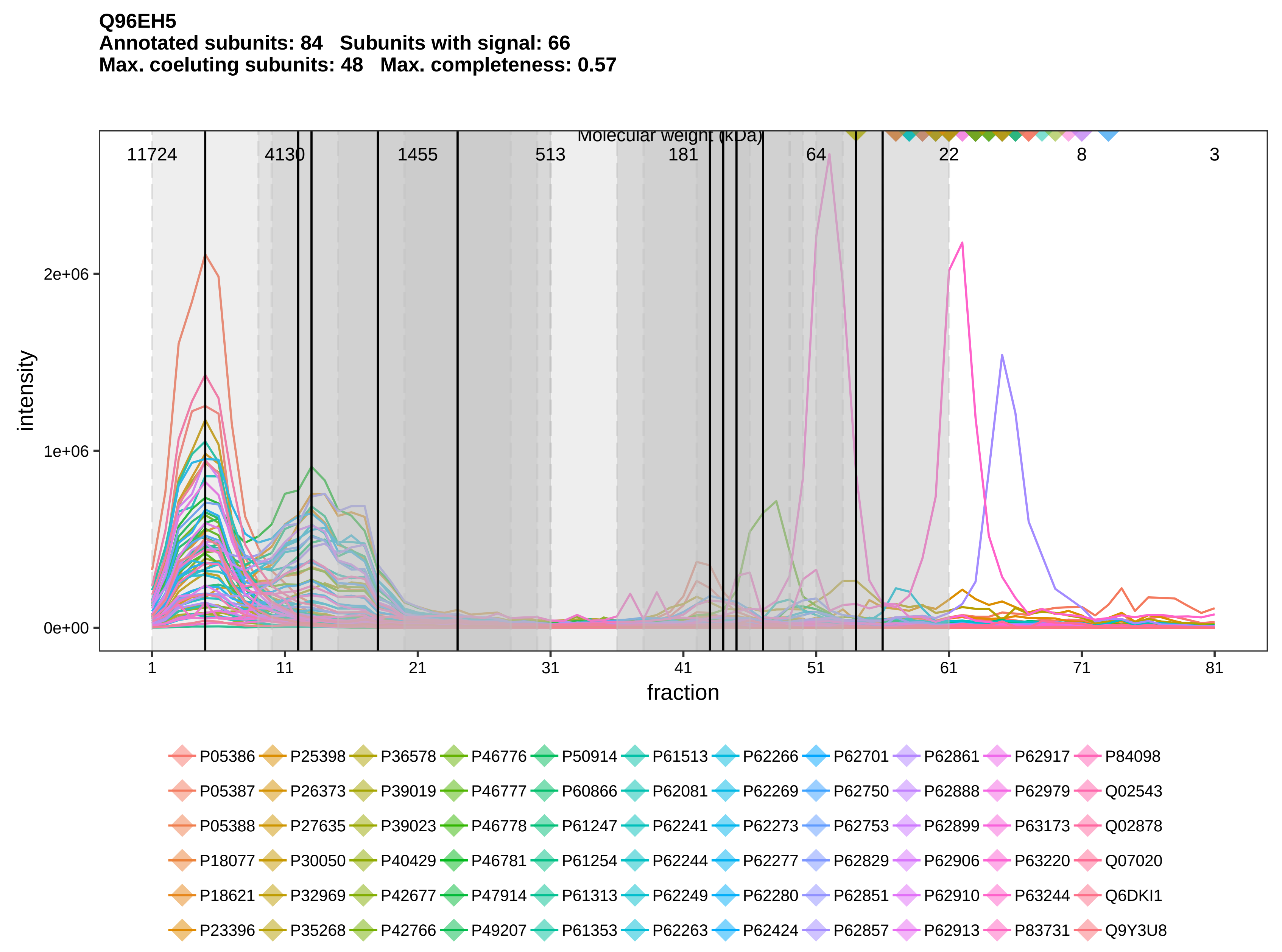
Q96EH5
Annotated subunits: 84 Subunits with signal: 66
Max. coeluting subunits: 48 Max. completeness: 0.57
Molecular weight (kDa)
11724
4130
1455
513
181
64
22
8
3
intensity
2e+06
1e+06
0e+00
fraction
1
11
21
31
41
51
61
71
81
P05386 P25398 P36578 P46776 P50914 P61513 P62266 P62701 P62861 P62917 P84098
P05387 P26373 P39019 P46777 P60866 P62081 P62269 P62750 P62888 P62979 Q02543
P05388 P27635 P39023 P46778 P61247 P62241 P62273 P62753 P62899 P63173 Q02878
P18077 P30050 P40429 P46781 P61254 P62244 P62277 P62829 P62906 P63220 Q07020
P18621 P32969 P42677 P47914 P61313 P62249 P62280 P62851 P62910 P63244 Q6DKI1
P23396 P35268 P42766 P49207 P61353 P62263 P62424 P62857 P62913 P83731 Q9Y3U8
